# Expression of VEGF in Peripheral Serum Is a Possible Prognostic Factor in Bone-Regeneration via Masquelet-Technique—A Pilot Study

**DOI:** 10.3390/jcm10040776

**Published:** 2021-02-15

**Authors:** Michael C. Tanner, Sonja Boxriker, Patrick Haubruck, Christopher Child, Fabian Westhauser, Christian Fischer, Gerhard Schmidmaier, Arash Moghaddam

**Affiliations:** 1 Center for Orthopedics, Trauma Surgery and Paraplegiology, Heidelberg University Hospital, 69118 Heidelberg, Germany; patrick.haubruck@med.uni-heidelberg.de (P.H.); christopher.child@med.uni-heidelberg.de (C.C.); fabian.westhauser@med.uni-heidelberg.de (F.W.); Christian.fischer@med.uni-heidelberg.de (C.F.); gerhard.schmidmaier@med.Uni-heidelberg.de (G.S.); 2 Center of Orthopedics, Trauma & Sports medicine, Aschaffenburg-Alzenau Hospital, 63739 Aschaffenburg, Germany; sonja.boxriker@klinikum-ab-alz.de (S.B.); arash.moghaddam@klinikum-ab-alz.de (A.M.)

**Keywords:** non-union, Masquelet-technique, induced membrane, bone regeneration, VEGF

## Abstract

Two-step Masquelet-technique established a new procedure in the treatment of osseous defects, addressing prerequisites postulated by the “diamond concept”. Increase in blood perfusion and growth factors are enhanced by the “Masquelet-membrane”. To describe this, we measured serum levels of Vascular Endothelial Growth Factor (VEGF) of patients with atrophic non-unions of long bones undergoing Masquelet-technique. From over 500 non-union patients undergoing Masquelet-technique with prospective follow-up we randomly selected 30 patients. 23 were included, 7 lost to follow-up or excluded because of incomplete data. Serum was drawn at specified intervals before and after surgery. Patients were followed for at least 6 months after step 2. Classification into both groups was performed according to radiological results and clinical outcome 6 months after step 2. Concentration of VEGF in patients’ serum was performed via ELISA. 14 achieved osseous consolidation (responder group), 9 cases did not (non-responder). Responders showed a significant increase of serum-VEGF in the first and second week when compared to the preoperative values of step 1. Non-responders showed a significant increase of VEGF in the second week after Steps 1 and 2. Comparison of groups showed significantly higher increase of serum-VEGF week2 after step 1 and preoperative to step 2 for responders. Results show one possibility of illustrating therapeutic progress by monitoring growth factors and possibly allowing prognostic conclusions thereof. This might lead to a more targeted treatment protocol.

## 1. Introduction

The delayed or non-union of long bones still poses a great challenge in trauma surgery. The complex physiological process of osseous healing may be interrupted by numerous factors [1,2,3,4], so that in general 10% and for high-risk groups up to 30% of patients may develop a non-union [5]. Development of non-union diminishes outcome and incurs immense socio-economic losses due to extensive therapy and unemployment of affected patients [6,7].

The so-called “Diamond Concept” describes the prerequisites for bone regeneration: Besides sufficient mechanical stability, osteoinductive scaffolds, osteogenetic cells and growth factors, perfusion plays a major role [8,9]. A so-called “biological chamber” has been proposed, which offers the optimal environment for the regeneration of bone [10].

In the past few years, the treatment of non-unions has been expanded by numerous techniques, including the Masquelet- or induced-membrane technique, with which three of the five prerequisites of the Diamond-Concept (osteogenetic cells, growth factors and perfusion) are fulfilled. It is a two-step procedure used in bone defects of different quality and has shown to be a viable choice in the reconstruction of long-distance bone defects and infects [11,12,13]. Described for the first time by Frenchman A.C. Masquelet [14], the procedure includes an initial operation with radical debridement of the pseudarthrosis and filling of the subsequent defect with a PMMA-spacer, optionally containing specific antibiotics. This induces a local foreign-body-reaction in the form of a membrane, amongst whose characteristics are the secretion of growth factors like TGF-ß, BMP-2 and VEGF as well as its abundance of blood vessels [15,16]. Additionally, it prevents the resorption of the osseous graft [17] and acts as a biochamber for bone regeneration [18,19,20,21,22]. In a second operative procedure the spacer is carefully removed under the preservation of said membrane and the resulting space filled with bone material, growth factors, scaffolds and antibiotics, as deemed necessary. In some papers with diverse patient numbers and levels of evidence this has been successfully carried out in various scenarios [23,24,25,26,27,28,29,30,31,32,33].

The importance of Vascular Endothelial Growth Factor (VEGF)for bone regeneration is well documented. It is one of the key factors for angio- and bone genesis. Further effects of VEGF are the proliferation and differentiation of osteoblasts and endothelial cells as well as the conversion of cartilage to bone [34,35,36,37,38,39].

So far, systemic examination of growth factors regarding their postoperative course under Masquelet-therapy has not yet comprehensively been performed. The goal of this study was to document the course of serum levels of VEGF in patients undergoing treatment of long-bone non-unions via Masquelet-technique and to see if any differences can be found between successful and unsuccessful treatments.

## 2. Materials and Methods

### 2.1. Study Design

From April 2012 onward, all patients with an atrophic non-union of a long-bone of their lower limb were prospectively enrolled into this study. From this pool of patients, 30 were randomly selected for this study. Seven of these did not meet inclusion criteria due to insufficient follow-up or insufficient data collection. All patients had previously consented to partake in this study. The study was conducted according to the guidelines of the Declaration of Helsinki and approved by the ethics committee of the University of Heidelberg (S-532/2011).

Step-1 surgery consisted of exchange of metalwork, non-union debridement, gathering of microbiological samples and implantation of a Gentamicin-laced cement spacer. On average 55 days afterward, in the second step procedure the induced membrane was carefully opened, the spacer removed and the void filled with a combination of autologous bone (iliac crest, RIA from the femur or fibular graft), BMP-7 (3.3 mg Osigraft^TM^, eptotermin alpha, Stryker) and tricalciumphosphate (VITOSS, Stryker).

### 2.2. Sample Retrieval and Measurement of Serum Cytokines

**Figure 1:**
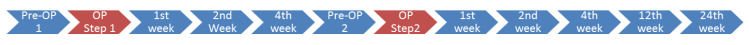
Standard protocol for retrieval of blood samples. Beginning of sampling preoperatively before Step 1 and Step 2 of Masquelet-Technique (Pre-OP 1 and 2) and in further course.

Patients had venous blood (7.5 mL Monovette, Sarstedt, Germany) drawn at the Center of Orthopedics, Trauma & Paraplegiology, Heidelberg University, Germany) in accordance with our standard protocol [40,41]. A modification was implemented due to the 2-step procedure (Figure 1). After retrieval, samples were immediately centrifuged (1000RPM, 10 min, 21 °C), serum pipetted and frozen at −80 °C.

Prior to analysis, samples were thawed for 2 h at room temperature. Vascular Endothelial Growth Factor (VEGF) was measured commercially available ELISA-kits (Quantikine^®^ ELISA Kit, R&D Systems, Minneapolis, MN, USA) and double-tested.

### 2.3. Clinical and Radiological Patient Outcome

All patients were clinically and radiologically evaluated preoperatively and up to 6 months after Step 2 of Masquelet-Procedure. Hereby conventional x-rays and, if deemed necessary, a CT-scan were performed as well as clinical outcome documented according to clinical protocol.

### 2.4. Data Analysis

Due to the not normal distribution of the data, non-parametric tests were used to compare samples. Statistical analysis for independent variables (different groups) was done with the Mann-Whitney-U-Test, dependent variables (within one group) were compared with the Wilcoxon-Signed-Rank-Test. The figures were illustrated as means and standard error of the mean.

### 2.5. Statistics

Statistical significance was determined according to the following criteria: significant *p* < 0.05 and very significant *p* < 0.01. Statistical analyses were done with SPSS Software (Deutschland GmbH, Ehningen, Germany). Graphs were created with Sigmaplot Software (Systat Software Inc., 1735 Technology Drive Suite 430, San Jose, CA, USA).

## 3. Results

Classification of patients into both study groups resulted according to radiological and clinical outcome six months after step 2 of Masquelet therapy. Successful therapy was defined as consolidation of three of four cortices of treated long bone, as evaluated by three consultant trauma surgeons of the Trauma department at the Center of Orthopedics, Trauma and Paraplegiology of Heidelberg University experienced in non-union surgery. Mechanical stability of the former non-union site demonstrated by full weight-bearing was also a necessity for determining successful therapy.

In order to assess patient smoker status, we compared patients’ Cotinin values with their statements on nicotine consumption. Cotinin values greater than 3.08 ng/mL were defined as active consumption in the near past and smoking status therefore recorded as positive [42].

### 3.1. Study Group 1: Responders

Our study group 1 Masquelet Responder consisted of fourteen patients (12 male and 2 female) with a mean age of 51.1 ± 10.82 years. The BMI was 27.1 ± 6.17. Four of fourteen patients were smokers. Three patients suffered from diabetes. Ten non-unions were in the tibial shaft (seven distal tibia, one multi-level-pseudarthrosis), in four cases in the femoral shaft. Their location was evenly distributed with seven cases left as well as right. Seven patients had a prior infection. Seven patients (50%) had an open fracture as intitial trauma. 5.5 operations had been performed in average after initial trauma (range 1 to 16 operations) prior to the masquelet procedures. In five cases flap or MESH coverage of soft tissues was necessary during treatment, in two of these cases after Step 2 of the Masquelet Technique. Three patients had no intervention before Masquelet Technique, respectively their non-union. Two patients were previously treated with an Ilizarov frame, in four cases grafting with cancellous bone had previously been performed. One patient had received BMP-7 prior to Masquelet Step 1. The mean time between step one and step two in Masquelet-technique was 52 days. Eight patients were stabilized via plate osteosynthesis, six with an intramedullary nail. Of these, three received a total ankle arthrodesis with a retrograde ETN-Protect Nail. Autologous bone and stem cells were harvested via RIA in six patients, five additionally received cancellous bone from the iliac crest and in one case we added a free fibula transplant. In two cases only iliac crest cancellous bone without RIA-material was transplanted (see Table 1).

**Figure 2:**
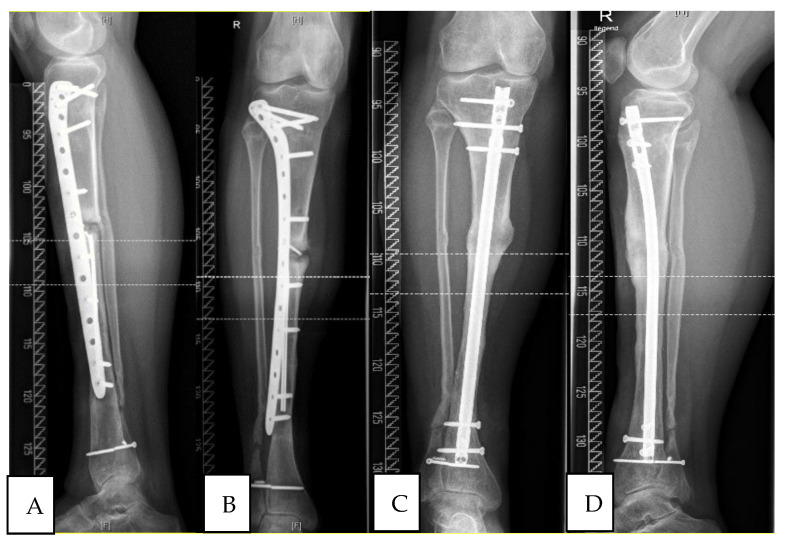
Atrophic tibial non-union (responder) initially treated with locking plate, converted to tibial nail according to diamond-concept, consolidation at 12 months post-op.(**A**) lateral view pre-OP; (**B**) ap-view pre-OP; (**C**) ap-view post-OP (**D**) lateral view post-OP.

All patients of this group showed radiological signs of consolidation 6 months after step 2 of Masquelet-technique (see Figure 2). Mechanical stability enabled full weight bearing.

### 3.2. Study Group 2: Non-Responder

A total number of nine patients (2 male and 7 female) (see Table 2) were included in this group. The mean age was 60.7 ± 13.41 and the average BMI 29.80 ± 6.20. Three of nine patients were smokers. Two patients suffered from diabetes. Six non-unions were in the femur (67%), only two in the tibia and one in the humerus. In five cases the right side was affected by non-union. Five of nine patients had an infection. Two of nine patients had an open fracture and needed a soft tissue replacement before Masquelet Technique. The mean number of operations after trauma was 7.2 (range 1 to 17 operations). In 5 cases an autologous bone graft had previously been applied and two patients had been treated with BMP-7 prior to Masquelet. The mean time between step one and step two in Masquelet-technique was 60 days. Internal fixation was achieved in five patients via a plate, in four patients with an intramedullary nail. Autologous bone graft was applied with RIA in five cases.

**Figure 3:**
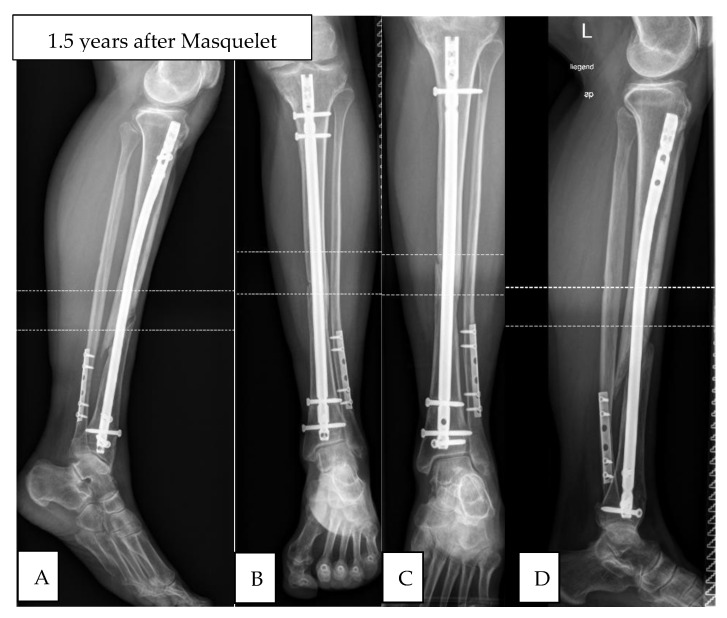
Atrophic tibial non-union (non- responder) initially treated with tibial nail, re-reamed and nailed according to diamond-concept, no consolidation at 12 months post-op. (**A**) lateral view pre-OP; (**B**) ap-view pre-OP; (**C**) ap-view post-OP; (**D**) lateral view post-OP.

Three of the nine patients in this group had revision surgeries within six months due to lack of consolidation or impending implant failure. The other six showed no signs of consolidation of their nonunions (see Figure 3). 

### 3.3. Serum Cytokine Levels

The changes of concentration of Vascular Endothelial Growth Factor over time are depicted as arithmetic means.

#### 3.3.1. Study Group 1: Responders

The preoperative mean concentration of VEGF in the study group Responder measured 628.05 ± 79.99 pg/mL and was defined as a reference. During the postoperative course after Step 1 significant elevated serum levels could be measured both in the first week (874.52 ± 124.99 pg/mL, *p* = 0.022) and the second week (1259.16 ± 111.50 pg/mL, *p* = 0.005). The second week’s value was also the maximum value in the development of VEGF.

After a decline in values up to Step 2 to a minimum of 710.72 ± 114.95 pg/mL in week 4, values again rose to 990.81 ± 126.29 pg/mL (*p* = 0.09) 1 week after Step 2 and to 1199.12 ± 132.53 pg/mL (*p* = 0.009) in week two.

**Figure 4:**
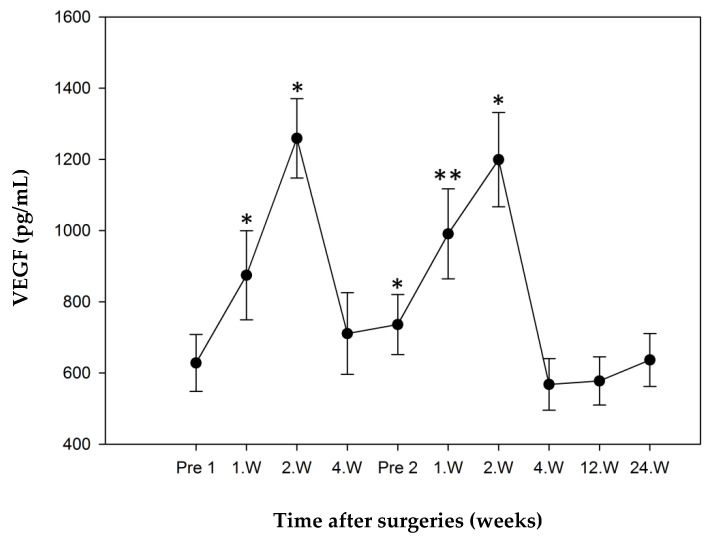
VEGF mean concentrations over time in responder group (error bars: standard error of the mean, * significant *p* < 0.05, ** very significant *p* < 0.01), all values compared to Pre 1 (preoperative value before Step 1). VEGF levels were significantly higher in week 1 and 2 after step 1 and 2 and preoperative before step 2. Maximum concentration of VEGF at second week after step 1.

The minimum value measured (567.95 ± 72.70 pg/mL) was seen 4 weeks after Step 2 surgery. All values measured in week 4 as well as the third and 6th month were not elevated in significant amounts to the preoperative reference value (Figure 4).

#### 3.3.2. Study Group 2: Non-Responder

**Figure 5:**
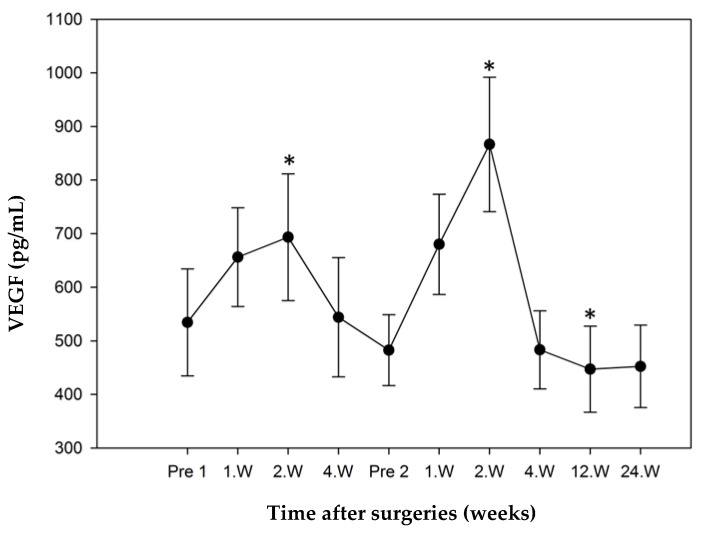
VEGF mean concentrations in non-responders over time (error bars: standard error of the mean, * significant *p* < 0.05), all values compared to Pre 1 (preoperatve value prior to step 1). VEGF levels were significant higher in the second week after step 1 and step 2. Maximum concentration of VEGF at second week after step 2.

The mean concentration of VEGF in the Non-responder study group measured 534.54 ± 99.83 pg/mL preoperatively and was also defined as reference. During the postoperative course after Step 1 there was an insignificant increase in VEGF to 656.10 ± 92.13 pg/mL (*p* = 0.18) in the first week, followed by a significant increase to 693.40 ± 118.25 pg/mL (*p* = 0.066). After an initial decrease to levels below the preoperative level just before Step 2 (482.64 ± 66.14 pg/mL), they rose significantly in week 2 following Step 2 surgery to a maximum of 866.63 ± 125.43 pg/mL (*p* = 0.018). All further values showed no statistical significance. The minimum of 452.35 ± 77.01 pg/mL was reached six months after Step 2 (Figure 5).

#### 3.3.3. Comparison of “Responders” and “Non-Responders”.

**Figure 6:**
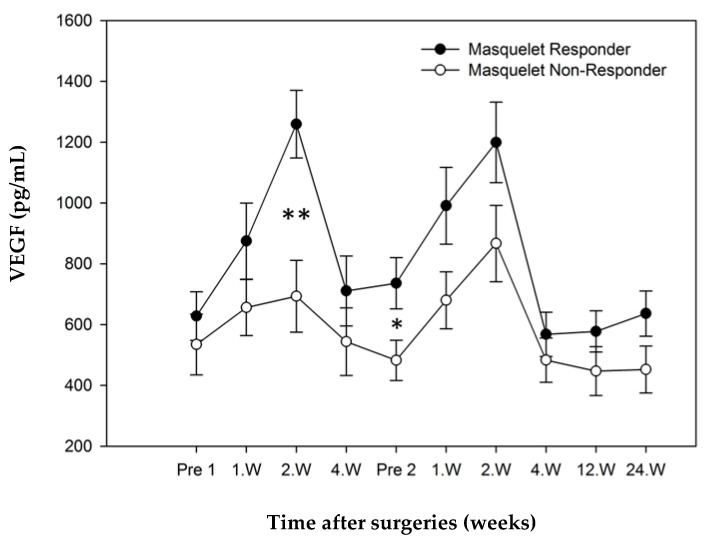
VEGF mean concentrations over time (error bars: standard error of the mean, * significant *p* < 0.05, ** very significant *p* < 0.01). Significant differences between both study groups in week 2 after step 1 and preoperative step 2.

During the whole course of observation, VEGF values for “responders” were higher than those of “non-responders”. Both groups showed peak values 1–2 weeks after respective surgeries. A significantly higher value could be seen in the responder group in the second week following step 1 (*p* = 0.005). Additionally, this group showed a significantly elevated value immediately before step 2 surgery (*p* = 0.044). The development of VEGF showed no significant values after step 2 surgery (Figure 6).

## 4. Discussion

In this prospective pilot study, we examined the course of VEGF serum concentration in patients with a long bone nonunion treated via Masquelet-Technique. To our knowledge (PubMed search for “VEGF and nonunion”), this is the first study to do so. We employed the standardized protocol from our previous studies [2,43], which was modified to accommodate the two-step procedure. As this study builds on methods showing the development of growth factors in nonunion therapy, we feel it might show promise in therapy evaluation and possible prognostic value.

Both study groups show a peak in concentration two weeks after step 1 and decline thereafter. We assume this is due to increased angiogenesis in the initial phase of induction of the Masquelet-membrane. The corresponding increase 2 weeks after step 2 is probably the consequence of the implantation of autologous bone and BMP-7. Studies of VEGF in consolidation after fractures showed similar courses [44,45]. Both studies showed a peak in VEGF concentration two weeks after fracture occurrence, with a gradual decline in further samples. Patients undergoing callus distraction procedures also examined VEGF concentrations in peripheral serum, showing similar increases two weeks after beginning of therapy [46]. Authors believe this is due to local angiogenesis secondary to callus distraction.

All these studies examined healthy test subjects [44,45]. Patients with fractures or undergoing callus distraction showed significantly higher concentrations of VEGF than the control groups. Whilst we were not able to employ a dedicated control group, the courses of VEGF concentration are similar to these previous studies and we are only comparing relative changes, not absolute values. Nonetheless, in further follow-up studies, such a control-group will be included.

VEGF plays a decisive role in the cascade of fracture healing, showing local and systemic increases [47]. In normal fracture healing, the fracture hematoma acts as a reservoir for growth factors [47]. We suppose that in Masquelet procedure, the membrane adopts this function and influences VEGF concentration.

The histological examination of human Masquelet membrane by Aho et al. [48] showed a chronological concordance of growth factor concentration and perfusion of the membrane. One month after implantation of the spacer, serum levels of growth factors were at their highest concentration, as was vascularization. Samples taken two months after implantation showed less than 40% of initial expression of VEGF, three months after implantation a decline in vascularization of more than 60% was detected. This is in accordance with our findings in serum concentration.

Henrich et al. [49] examined the membrane in rats and showed that the activity concerning vascular and osseous growth was highest from two to four weeks post intervention and began to decline after six weeks. Another animal study performed by Pelissier et al. [15] showed high concentrations of VEGF in the membrane two weeks after spacer implantation, albeit subcutaneous and not orthotopic. Under the assumption that local changes in the membrane influence the concentration of VEGF, our findings are in concordance with these histological and immunohistochemical results.

The comparison of both study groups showed significantly higher values in the responder group two weeks after step one and preoperative to step 2. These differences show chronological correlation with membrane induction, which seems to be a decisive factor due to its protection and nutrition of the graft.

The revelation of a prognostic factor (i.e., biomarker) in the often lengthy Masquelet therapy [50] would be an important tool in therapeutic planning, as previous studies and our own experience have shown that consolidation is often not apparent until up to nine months after step 2. As the definition of radiological consolidation is nonuniform amongst orthopedic & trauma surgeons [51], a prognostic marker in an early phase of the therapeutic regimen would prove valuable. The small size of and inhomogeneous composition of our collective does not yet allow classification of VEGF as a definitive biomarker. For this conclusion we need a larger collective. Nonetheless, this pilot study shows promise that further studies might support these initial results.

Shortcomings of this study are the small sample size and the composition of the collective. For this pilot study we randomly chose 30 individuals from our collective, thereby eliminating the possibility of matching patients. The heterogeneity of the small collective impeded examination for other possible influential factors. Striking was the percentage of female patients in the responder (2 of 14, 14%) and non-responder groups (7 of 9, 78%). 

Considering the fact that studies [52] have shown that VEGF is significantly lower in postmenopausal women, this distribution might be a confounding factor. On the other hand, age greater than 60 has not been shown to be a risk factor for non-union in larger cohorts Localization of nonunions also showed interesting distribution: the tibia was affected in 71% of responders (10 of 14) and 22% in non-responders (2 of 9).

Due to the promising results we aim to apply these methods to a greater collective in order to validate our results as much as possible. We would thereby also have the possibility of searching for other possible influential factors such as gender, prior illnesses or localization.

## 5. Conclusions

Under consideration of the limitations of this study it is not yet justifiable to employ the lowered VEGF values of the non-responder group as a prognostic factor. In the future, serum measurement of VEGF should be performed in a greater number of patients in order to first confirm these preliminary results and to define further possibly influential factors on VEGF secretion. Further examination of humane Masquelet-membrane is also necessary to further understand the biochemical processes involved in the initial phase of osseous reconstruction in Masquelet technique. Nonetheless, the results from this pilot study show promising results and first indications for establishing a prognostic marker in nonunion therapy. Further studies in this direction should be striven for.

## Figures and Tables

**Table 1:** Study Group Responder.

No.	Gender	Age	BMI	Localisation	Side	Pre-OPs	Previous Infection	Fixation	Bone Graft	Diabetes	Smoker	Cotinin
F001	M	72	22.8	Tibia	left	>10	yes	Nail	RIA, free fibula	no	no	positive
F006	M	45	19.8	Tibia	left	4	yes	Plate	RIA	no	yes	positive
F009	F	50	32.5	Femur	left	2	no	Nail	RIA	no	no	negative
F010	M	64	25.1	Tibia	right	3	yes	Nail	Iliac crest	yes	yes	negative
F012	M	30	26.7	Tibia	right	>10	yes	Nail	RIA	no	no	positive
F013	M	51	29.9	Tibia	right	4	yes	Plate	RIA, iliac crest	no	no	negative
F021 *	M	59	22.8	Femur	left	4	no	Plate	Iliac crest	no	yes	positive
F025	M	53	29.7	Tibia	right	16	yes	Nail	RIA, iliac crest	yes	no	positive
F026	M	50	27.5	Femur	left	4	no	Nail	RIA	no	no	positive
F027	M	45	44.8	Femur	right	2	no	Plate	RIA	yes	no	negative
F028	F	38	23.1	Tibia	right	4	no	Plate	RIA, iliac crest	no	no	negative
F029	M	55	21.9	Tibia	left	3	no	Plate	RIA, iliac crest	no	yes	positive
F030	M	60	25.9	Tibia	right	1	no	Plate	RIA, iliac crest	no	no	negative
F031	M	43	26.3	Tibia	left	10	yes	Plate	RIA	no	no	negative

Patient demographics: M, male; F, female; BMI, body mass index in kg/m^2^; Pre-OPs: All interventions at site since trauma; Previous Infection: current or prior to op; Bone graft: Autologous bone graft used in Step 2; Cotinin positive = Cotinin > 3.08 ng/mL; * Treatment with Osigraft^TM^ (OP-1, rhBMP-7) prior to Masquelet Technique.

**Table 2:** Study group Non-Responder.

No.	Gender	Age	BMI	Localisation	Side	Pre-OPs	Previous Infection	Fixation	Bone Graft	Diabetes	Smoker	Cotinin
F002	F	51	30.4	Femur	left	>10	yes	Plate	RIA, iliac crest	no	yes	positive
F003	F	57	22.2	Femur	left	17	yes	Nail	RIA	no	yes	positive
F004 *	F	60	39.2	Femur	right	3	No	Plate	RIA	no	no	negative
F005	F	73	37.5	Humerus	left	1	No	Plate	iliac crest	yes	no	negative
F007 *	M	44	25.2	Tibia	left	12	yes	Nail	RIA	no	no	negative
F008	F	71	29.7	Femur	right	14	yes	Nail	RIA, iliac crest	no	no	negative
F018	M	71	26.3	Tibia	right	4	yes	Nail	RIA	yes	no	negative
F023	F	41	34.6	Femur	right	2	No	Plate	RIA	no	yes	negative
F035	F	78	23.0	Femur	right	2	No	Plate	iliac crest	no	no	negative

Patient demographics: M, male; F, female; BMI, body mass index in kg/m^2^; Pre-OPs: All interventions at site since trauma; Previous Infection: current or prior to op; Bone graft: Autologous bone graft used in Step 2; Cotinin positive = Cotinine > 3.08 ng/mL; * Treatment with Osigraft^TM^(OP-1, rhBMP-7) prior to Masquelet Technique.

## Data Availability

The data presented in this study are available on request from the corresponding author.
